# Supplementary material for: Ubiquitin-Proteasome Modulating Dolabellanes and Secosteroids from Soft Coral Clavularia flava
Source: Mar Drugs. 2020 Jan 3;18(1):39. doi: 10.3390/md18010039 (PMC7024272; doi:10.3390/md18010039)
Supplement: Supplementary file 1 [file marinedrugs-18-00039-s001.pdf]

Supplementary Materials

Table of Contents 1

Figure S1. HRESIMS of **1** 2

Figure S2. **I**R spectrum of **1** 3

Figure S3. ^{1}H NMR spectrum (400 MHz) of **1** in $CDCl_3$ 4

Figure S4. ^{13}C NMR spectrum (100 MHz) of **1** in $CDCl_3$ 5

Figure S5. HSQC spectrum (400 MHz) o**f 1** in $CDCl_3$ 6

Figure S6. COSY spectrum (400 MHz) of **1** in $CDCl_3$ 7

Figure S7. spectrum (400 MHz) of **1** in $CDCl_3$ 8

Figure S8. NOESY spectrum (400 MHz) o**f 1** in $CDCl_3$ 9

Figure S9. HRESI of **5** 10

Figure S10. IR spectrum of **5** 11

Figure S11. ^{1}H NMR spectrum (400 MHz) of **5** in C_6D_6 12

Figure S12. ^{13}CNMR spectrum (100 MHz) of **5** in C_6D_6 13

Figure S13. HSQC spectrum (400 MHz) of **5** in C_6D_6 14

Figure S14. COSY spectrum (400 MHz) of **5** in C_6D_6 15

Figure S15. HMBC spectrum (400 MHz) of **5** in C_6D_6 16

Figure S16. NOESY spectrum (400 MHz) of **5** in C_6D_6 17

Figure S17. HRESIof **6** 18

Figure S18. IR spectrum of **6** 19

Figure S19. ^{1}H NMR spectrum (400 MHz) of **6** in C_6D_6 20

Figure S20. ^{13}CNMR spectrum (100 MHz) of **6** in C_6D_6 21

Figure S21. HSQC spectrum (400 MHz) of **6** in C_6D_6 22

Figure S22. COSY spectrum (400 MHz) of **6** in C_6D_6 23

Figure S23. HMBC spectrum (400 MHz) of **6** in C_6D_6 24

Figure S24. NOESY spectrum (400 MHz) of **6** in C_6D_6 25

Table S1. Evaluation of ED_{50} cytotoxicity of tested compounds. 26

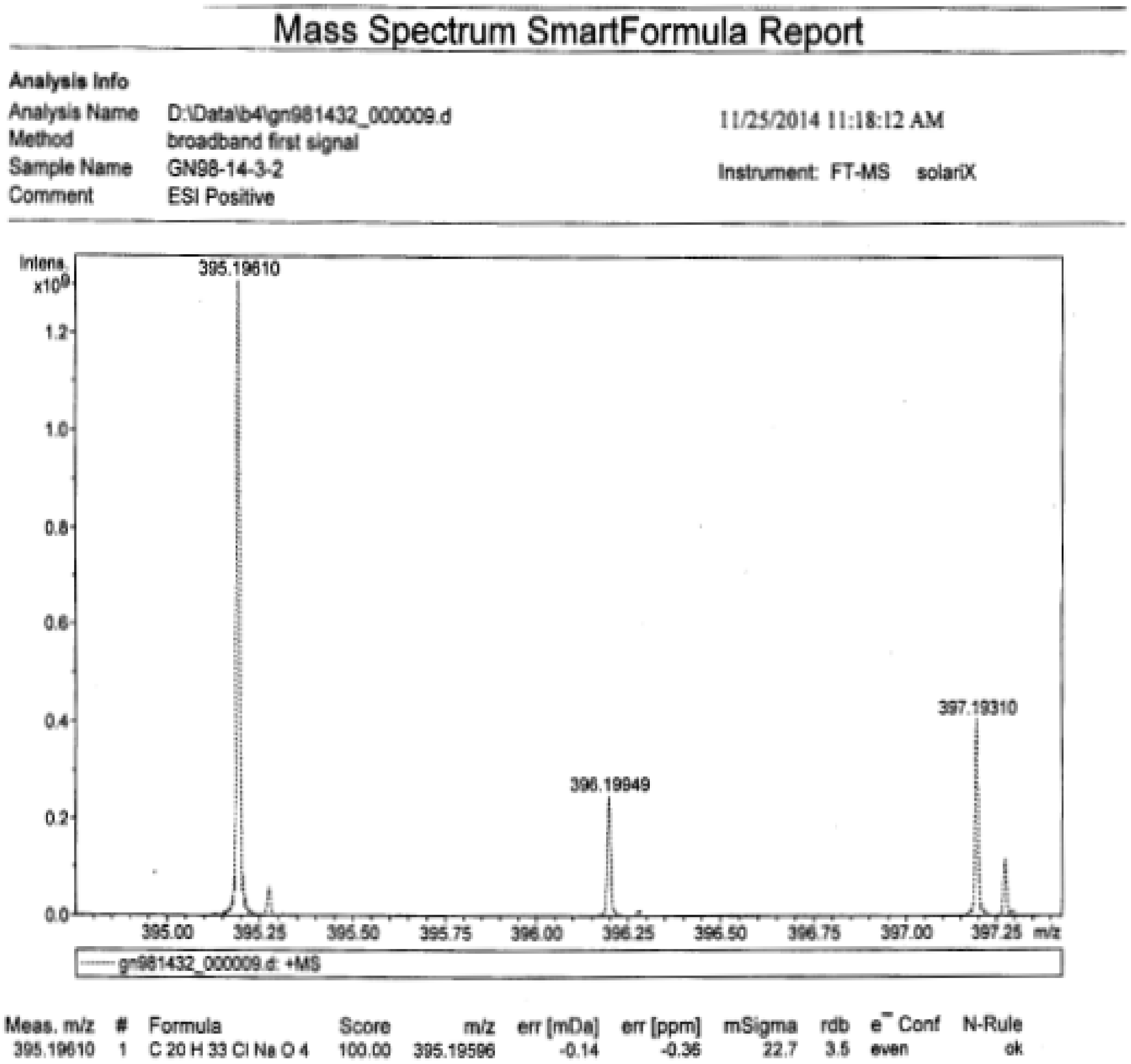

Mass Spectrum SmartFormula Report

Analysis Info

Analysis Name D:\Data\b4\gn981432_000009.d — 11/25/2014 11:18:12 AM
Method broadband first signal
Sample Name GN98-14-3-2 — Instrument: FT-MS solariX
Comment ESI Positive

Meas. m/z	#	Formula	Score	m/z	err [mDa]	err [ppm]	mSigma	rdb	e⁻ Conf	N-Rule
395.19610	1	C 20 H 33 Cl Na O 4	100.00	395.19596	-0.14	-0.36	22.7	3.5	even	ok

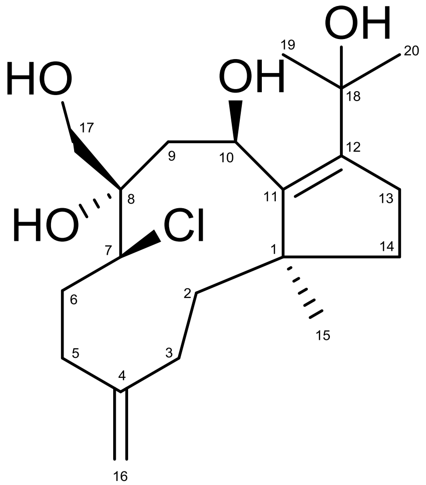

1

Figure S1. HRESIMS of **1**

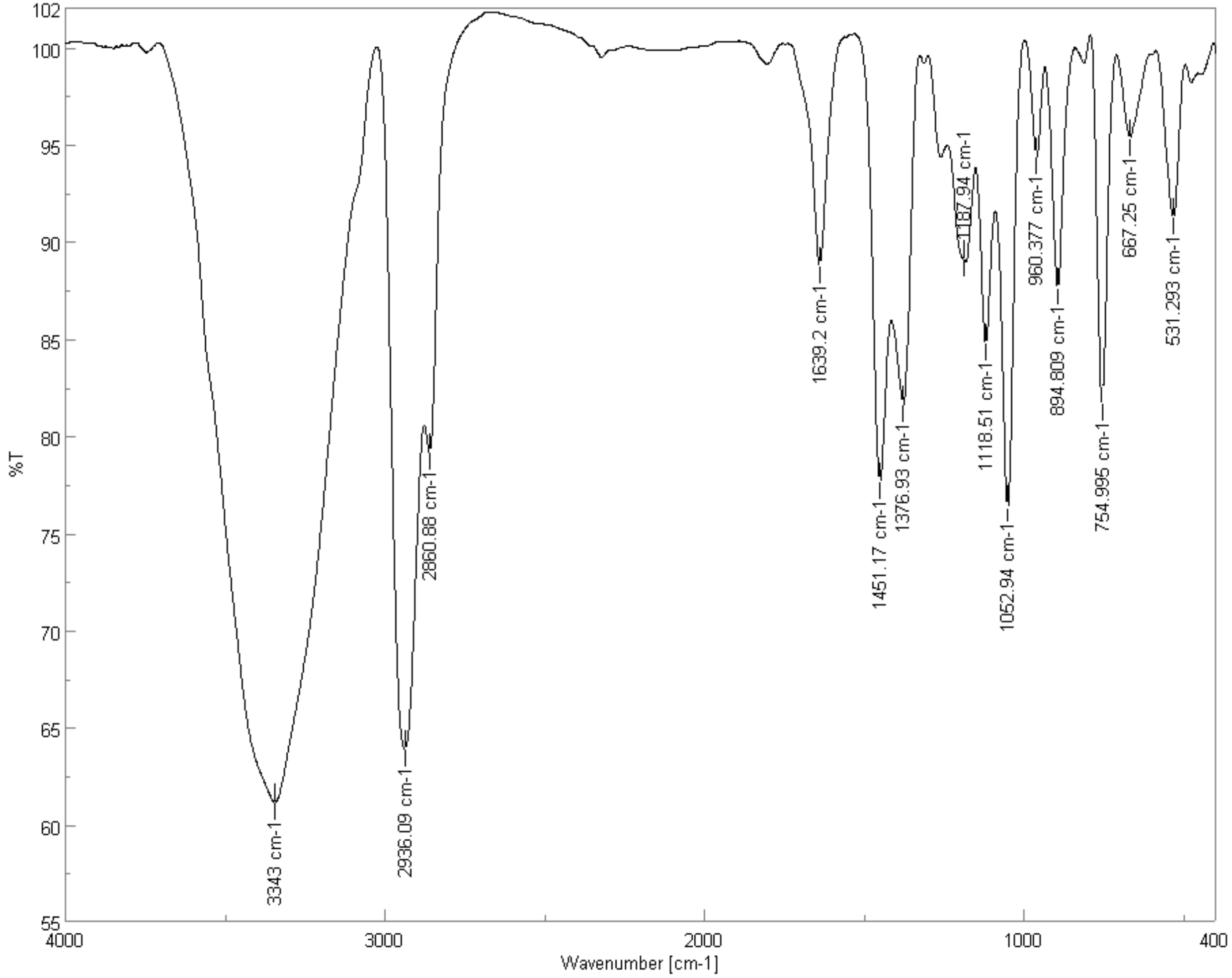

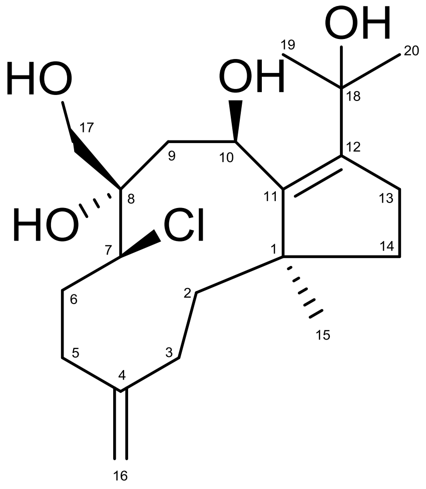

1

Figure S2. IR spectrum of **1**

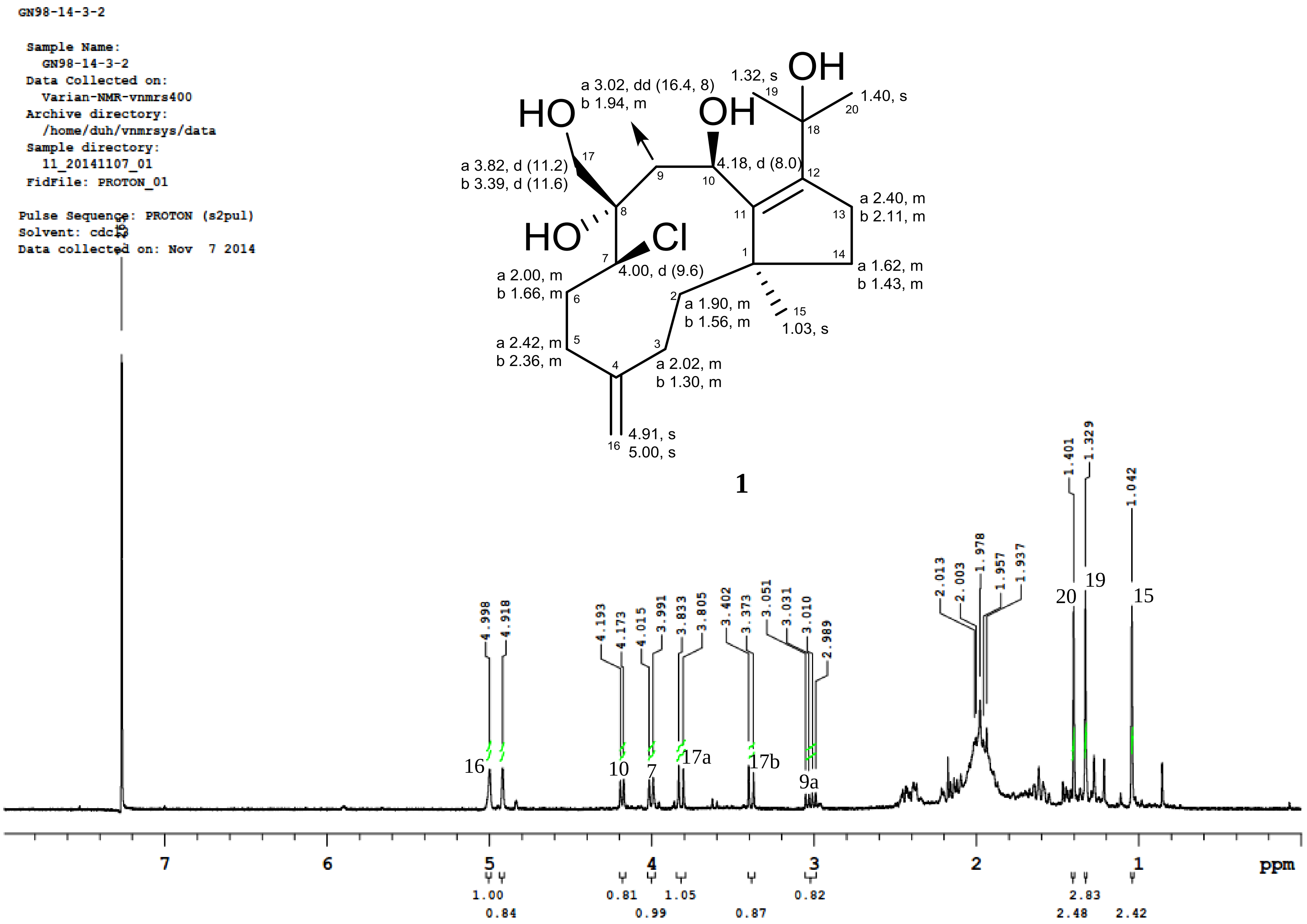

Figure S3. ^{1}H NMR spectrum (400 MHz) of **1** in $CDCl_3$

GN98-14-3-2

Sample Name:
GN98-14-3-2
Data Collected on:
Varian-NMR-vnmrs400
Archive directory:
/home/duh/vnmrsys/data
Sample directory:
GN98-14-3-2_20141104_01
FidFile: CARBON_01

Pulse Sequence: CARBON (s2pul)
Solvent: cdcl3
Data collected on: Nov 4 2014

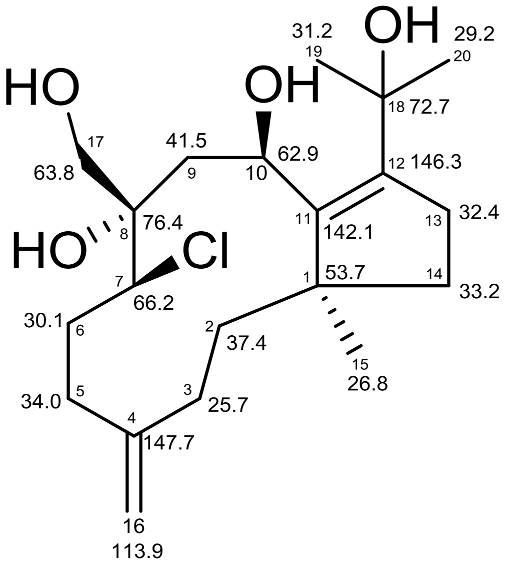

1

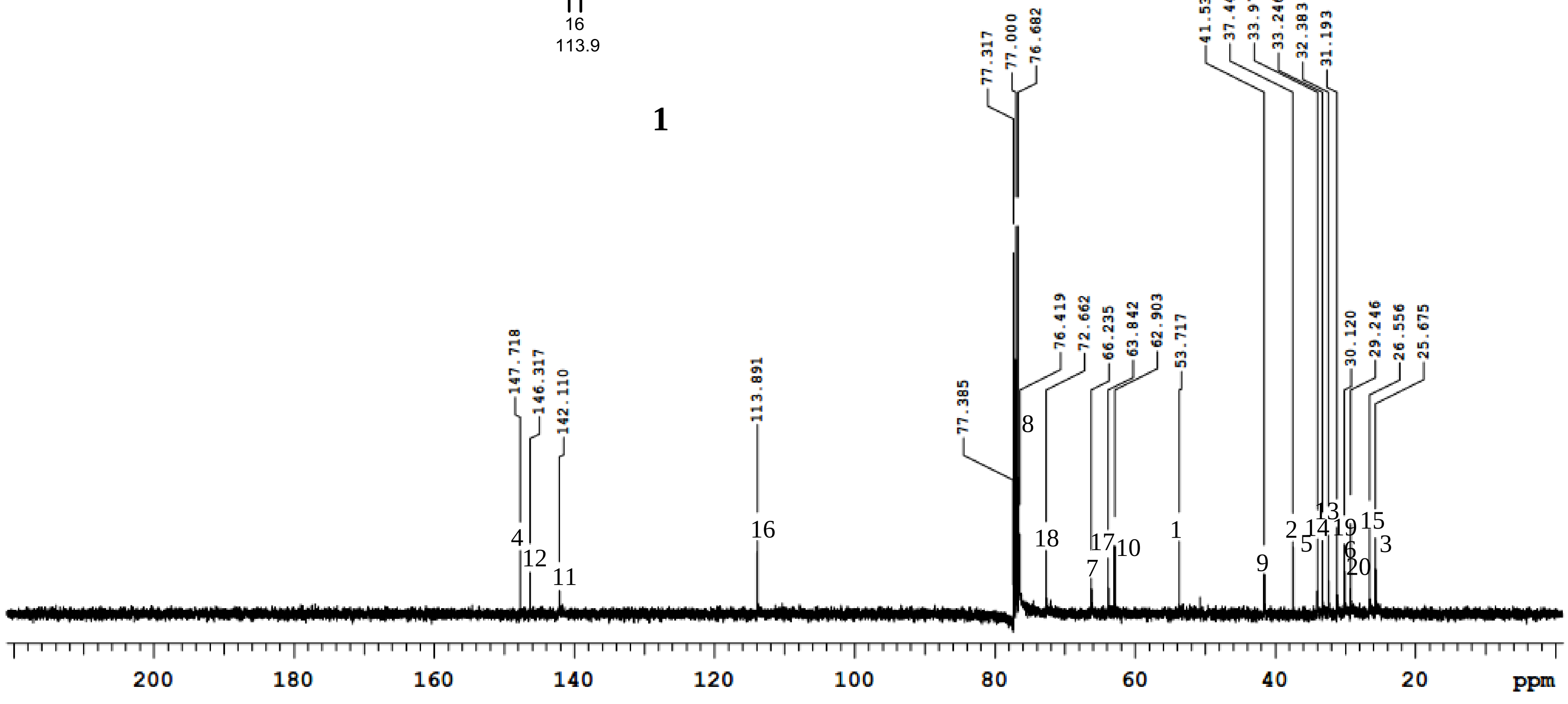

Figure S4. ^{13}C NMR spectrum (100 MHz) of **1** in $CDCl_3$

GN98-14-3-2

Sample Name:
GN98-14-3-2
Data Collected on:
Varian-NMR-vnmrs400
Archive directory:
/home/duh/vnmrsys/data
Sample directory:
GN98-14-3-2_20141104_01
FidFile: gHSQCAD_01

Pulse Sequence: gHSQCAD
Solvent: cdcl3
Data collected on: Nov 4 2014

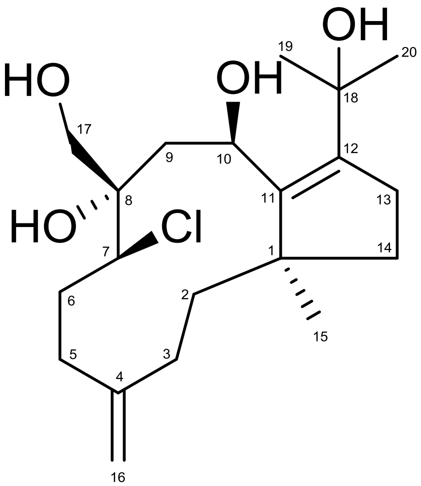

1

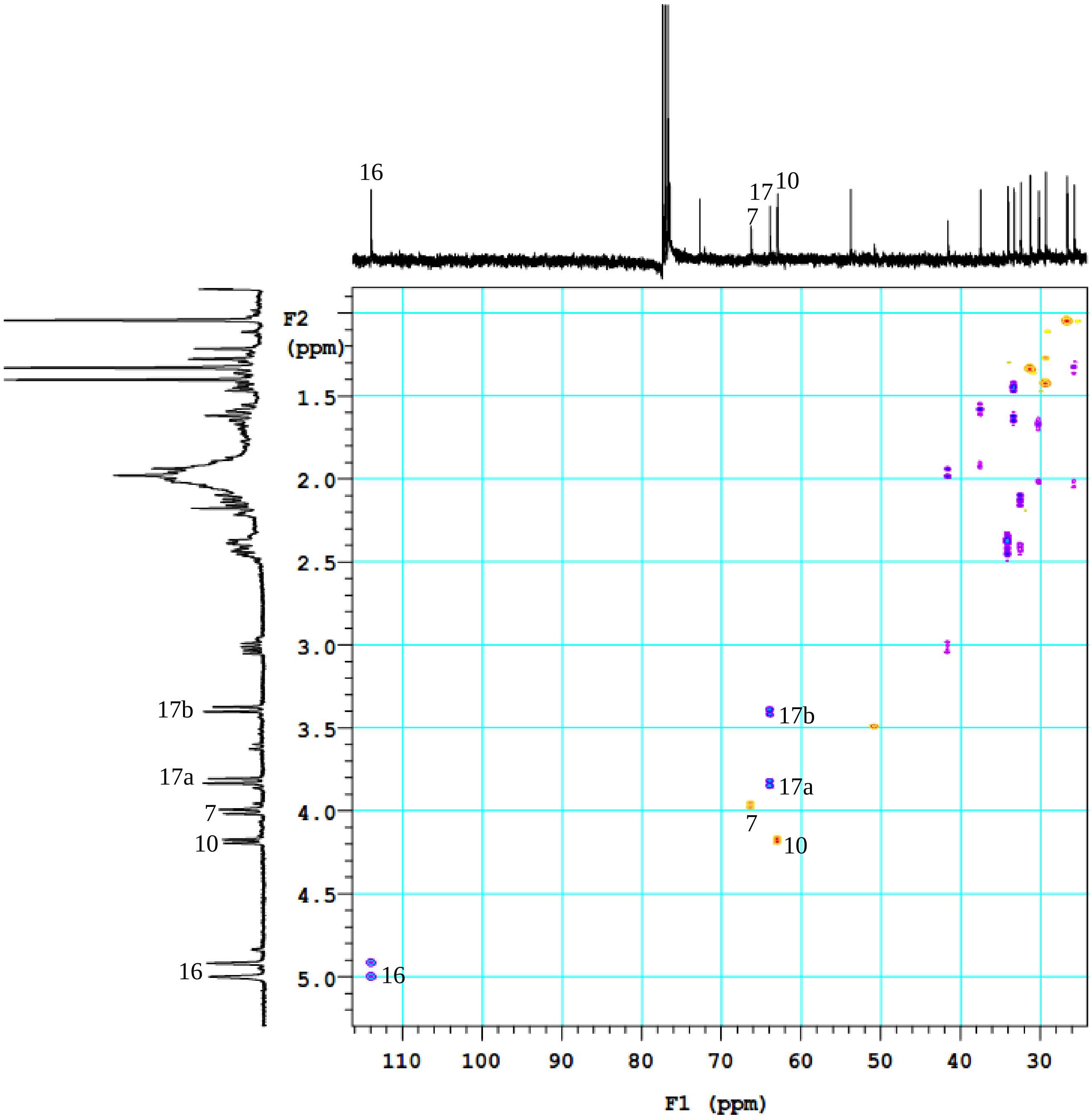

Figure S5. HSQC spectrum (400 MHz) of **1** in $CDCl_3$

GN98-14-3-2

Sample Name:
GN98-14-3-2
Data Collected on:
Varian-NMR-vnmrs400
Archive directory:
/home/duh/vnmrsys/data
Sample directory:
GN98-14-3-2_20141104_01
FidFile: gCOSY_01

Pulse Sequence: gCOSY
Solvent: cdcl3
Data collected on: Nov 4 2014

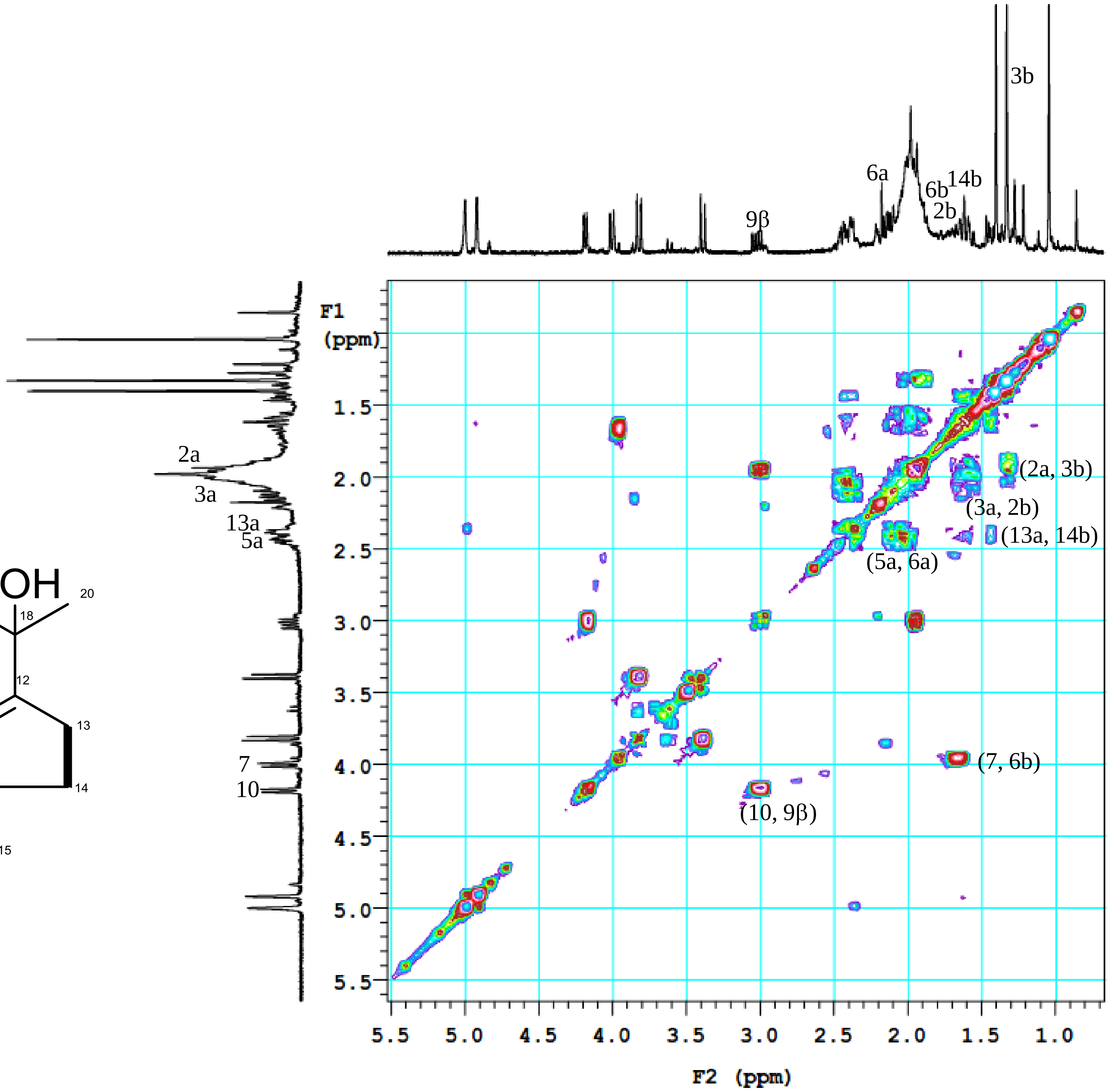

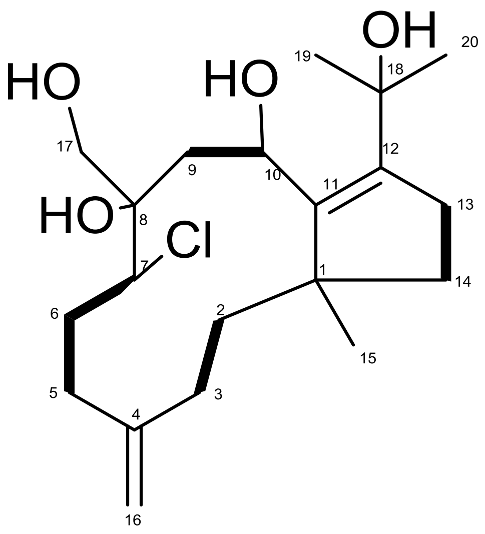

1

Figure S6. COSY spectrum (400 MHz) of **1** in $CDCl_3$

```
GN98-14-3-2

 Sample Name:
   GN98-14-3-2
 Data Collected on:
   Varian-NMR-vnmrs400
 Archive directory:
   /home/duh/vnmrsys/data
 Sample directory:
   GN98-14-3-2_20141104_01
 FidFile: gHMBCAD_01

Pulse Sequence: gHMBCAD
Solvent: cdcl3
Data collected on: Nov  4 2014
```

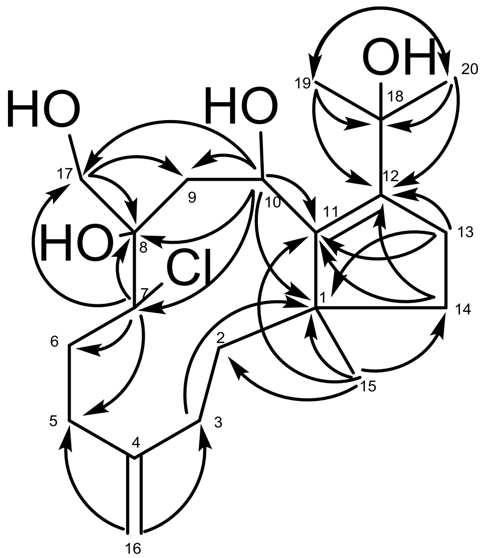

1

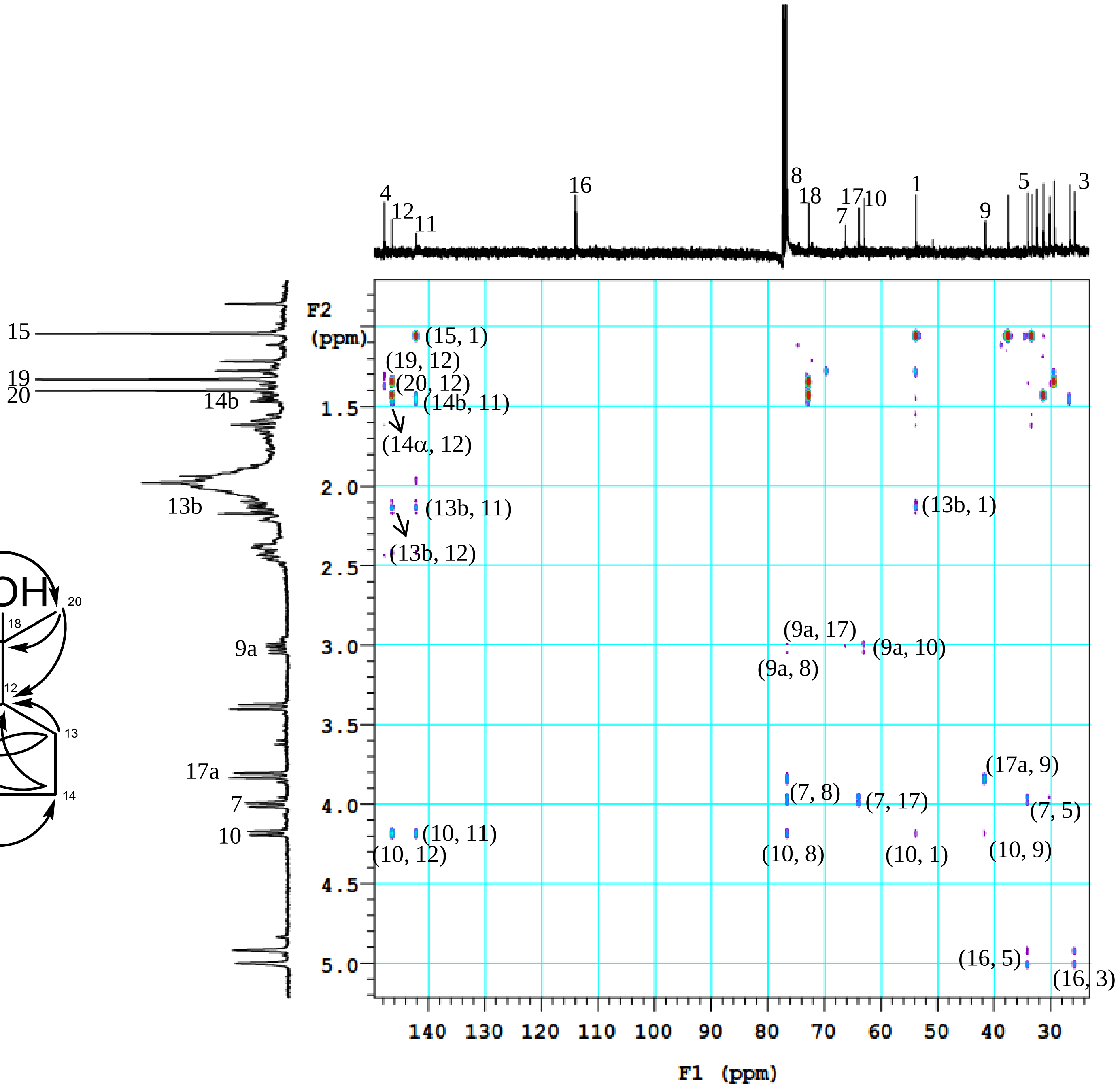

Figure S7. spectrum (400 MHz) of **1** in $CDCl_3$

GN98-14-3-2

Sample Name:
GN98-14-3-2
Data Collected on:
Varian-NMR-vnmrs400
Archive directory:
/home/duh/vnmrsys/data
Sample directory:
GN98-14-3-2_20141104_01
FidFile: NOESY_01

Pulse Sequence: NOESY
Solvent: cdcl3
Data collected on: Nov 4 2014

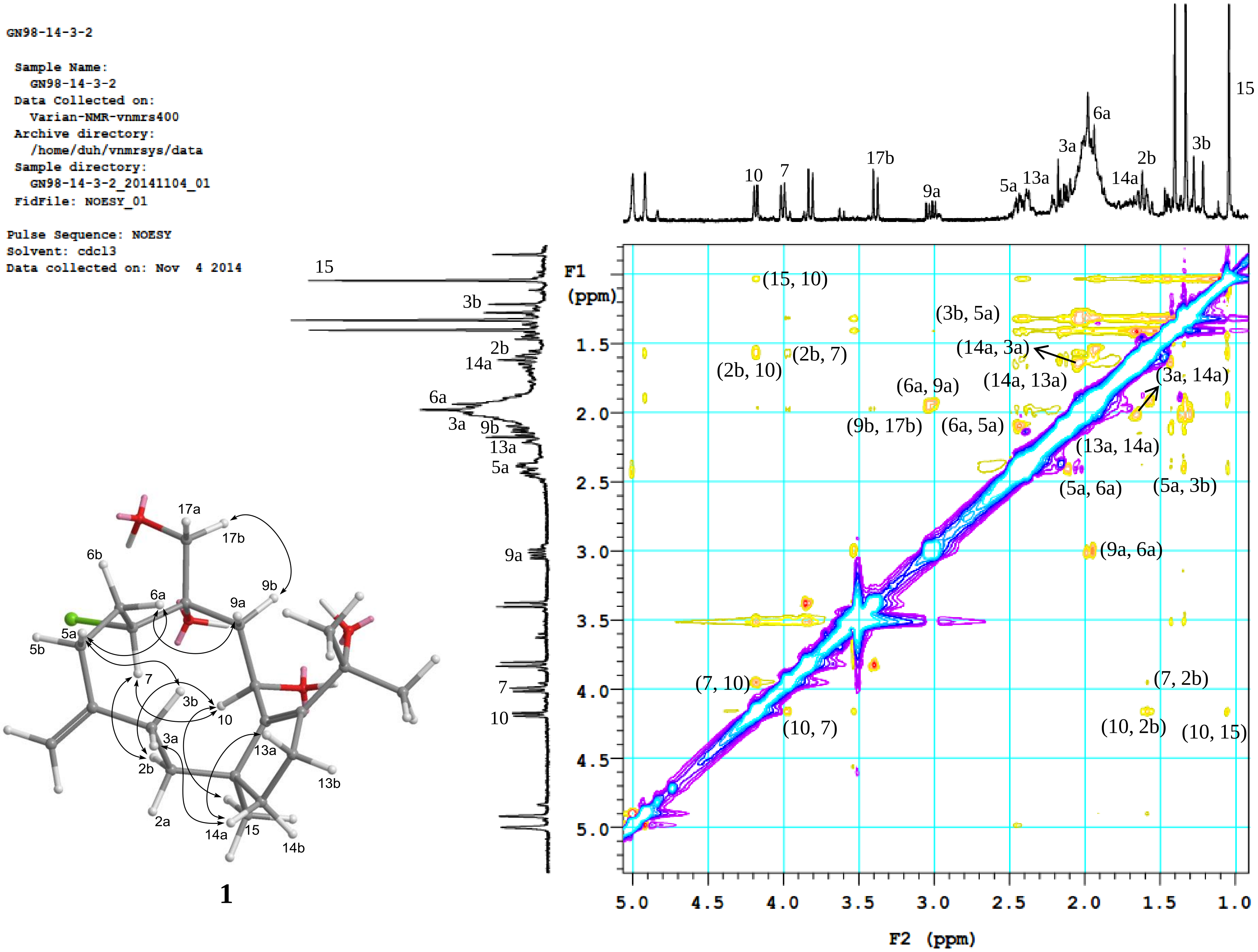

Figure S8. NOESY spectrum (400 MHz) of **1** in $CDCl_3$

Mass Spectrum SmartFormula Report

Analysis Info

Analysis Name	D:\Data\b4\gn9815371_000002.d	1/26/2015 2:28:03 PM
Method	broadband first signal	
Sample Name	GN98-15-3-7-1	Instrument: FT-MS solariX
Comment	ESI Positive	

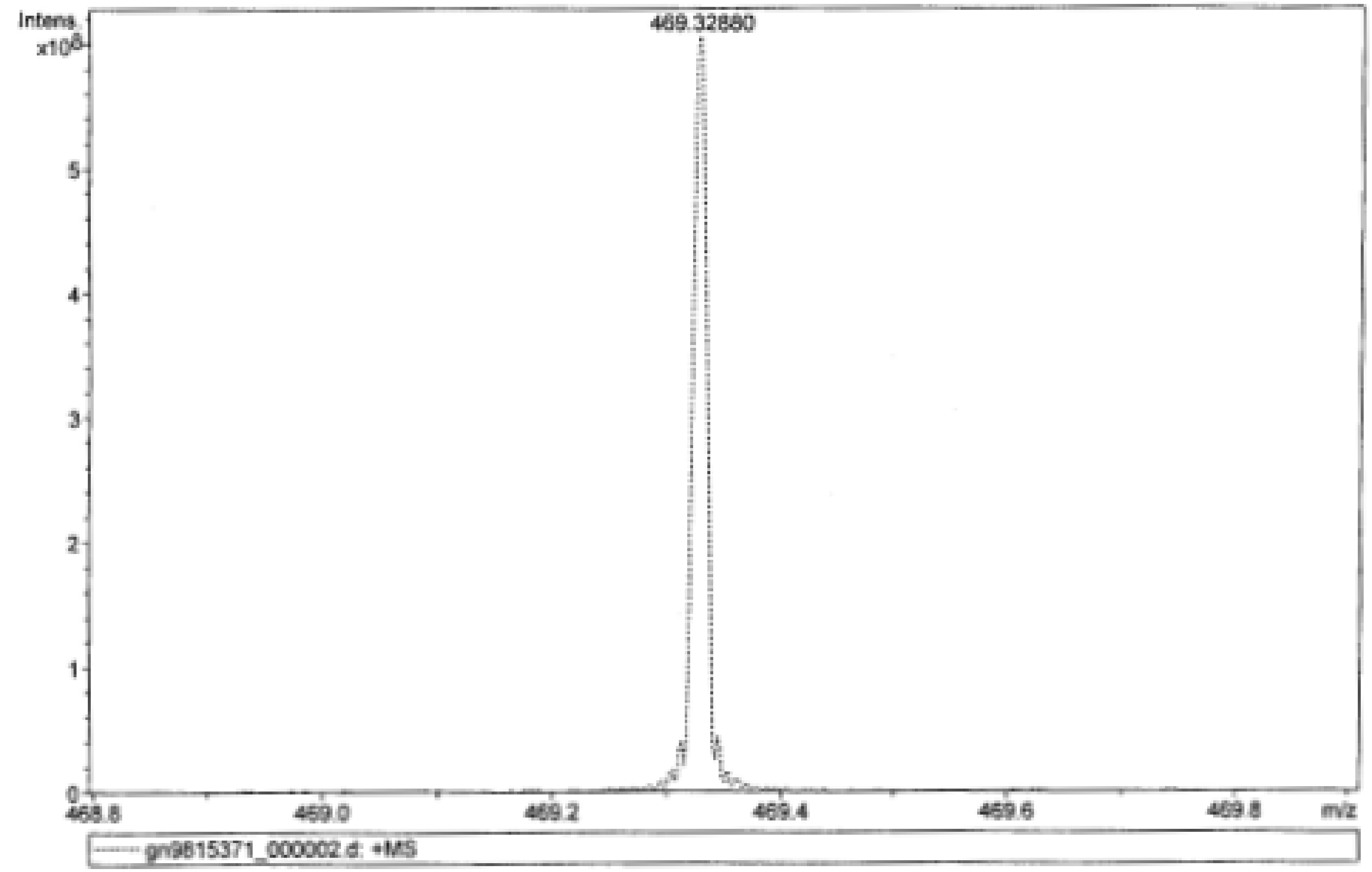

Meas. m/z	#	Formula	Score	m/z	err [mDa]	err [ppm]	mSigma	rdb	e⁻ Conf	N-Rule
469.32880	1	C 28 H 46 Na O 4	100.00	469.32883	0.03	0.06	4.2	5.5	even	ok

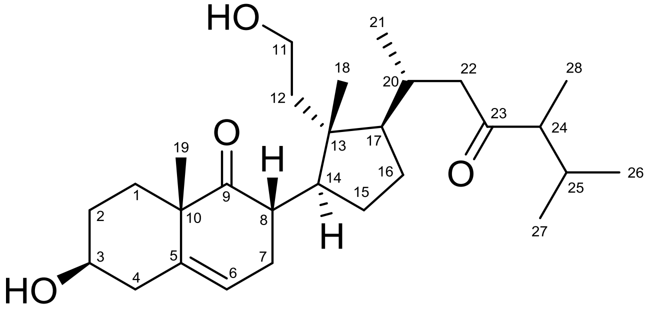

5

Figure S9. HRESIof **5**

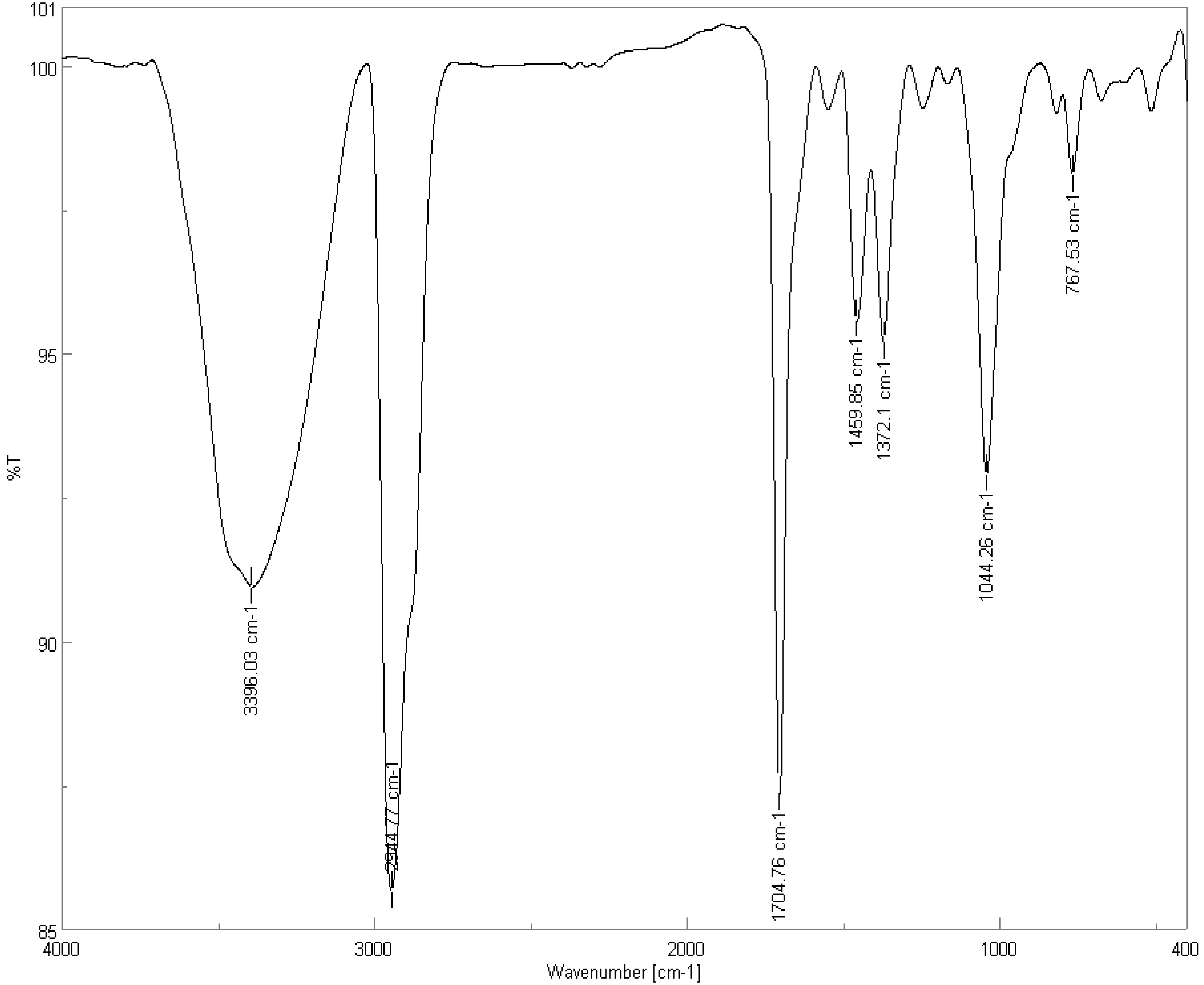

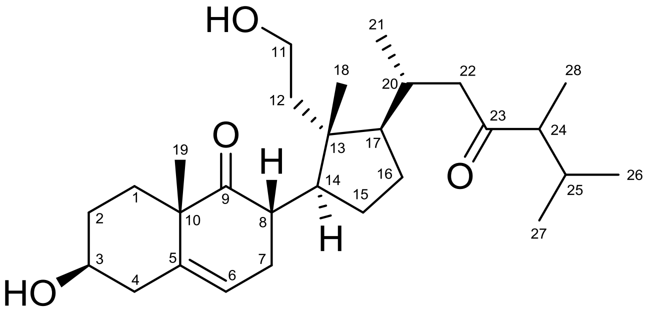

5

Figure S10. IR spectrum of **5**

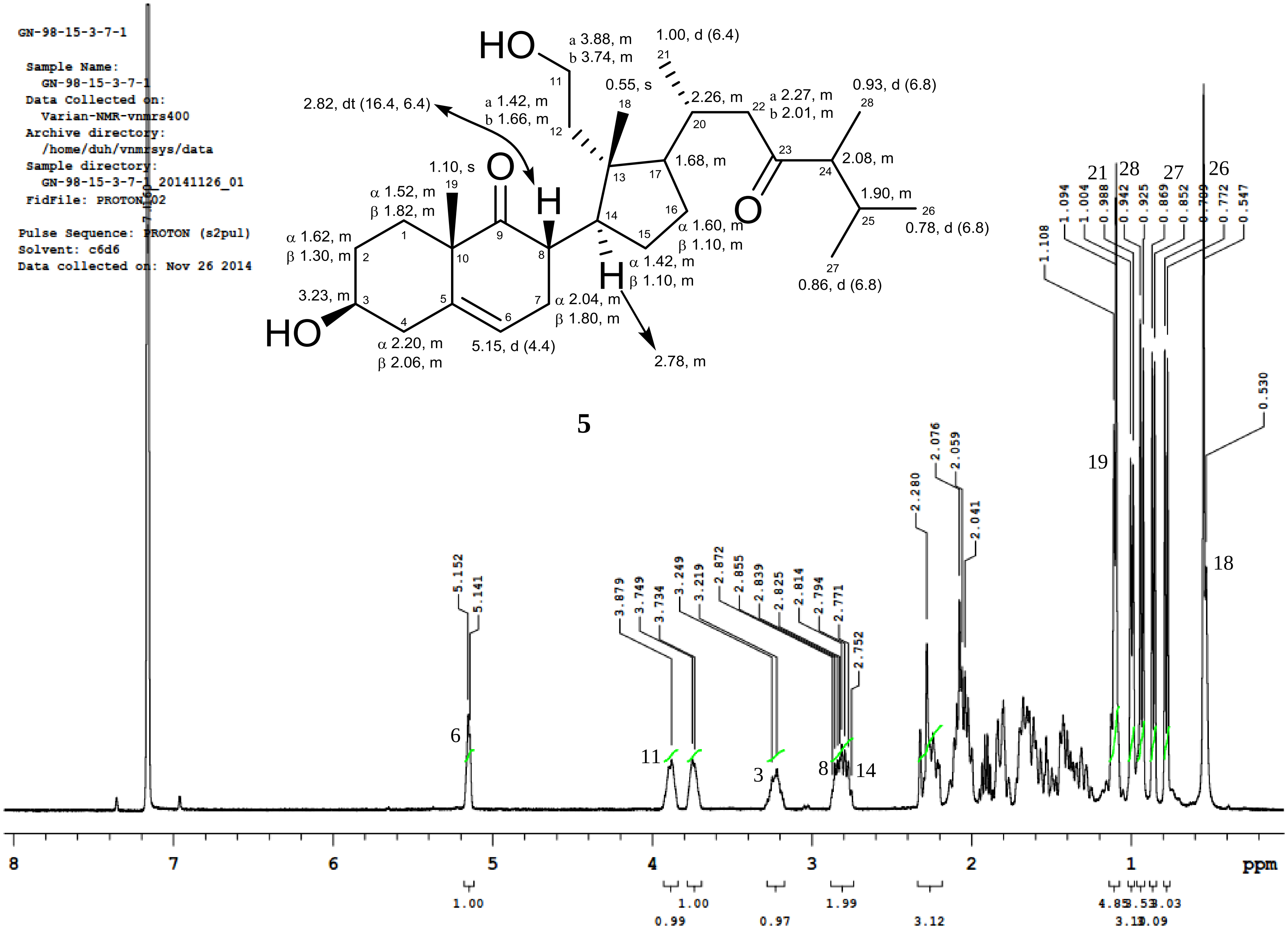

Figure S11. ^1H NMR spectrum (400 MHz) of **5** in C_6D_6

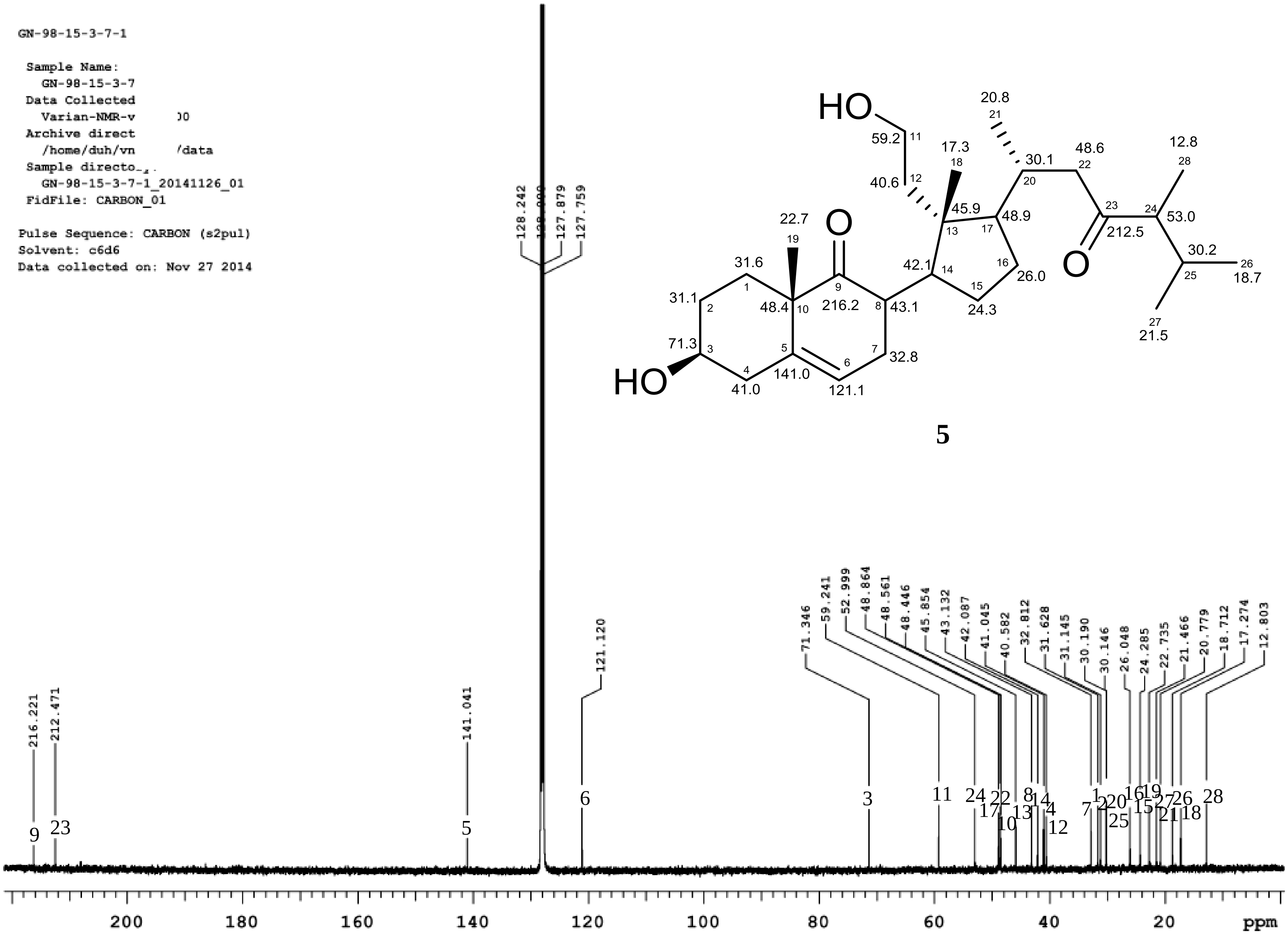

Figure S12. ^{13}CNMR spectrum (100 MHz) of **5**in C_6D_6

GN-98-15-3-7-1

Sample Name:
GN-98-15-3-7-1
Data Collected on:
Varian-NMR-vnmrs400
Archive directory:
/home/duh/vnmrsys/data
Sample directory:
GN-98-15-3-7-1_20141126_01
FidFile: gHSQCAD_01

Pulse Sequence: gHSQCAD
Solvent: c6d6
Data collected on: Nov 27 2014

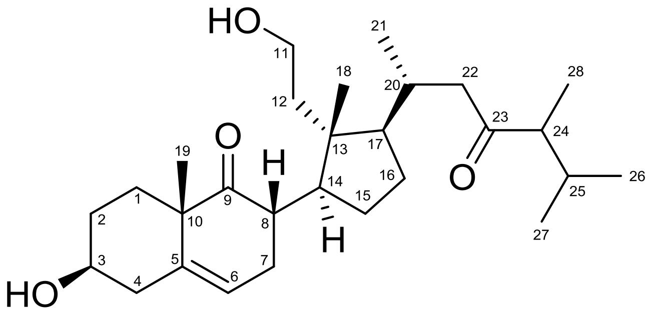

5

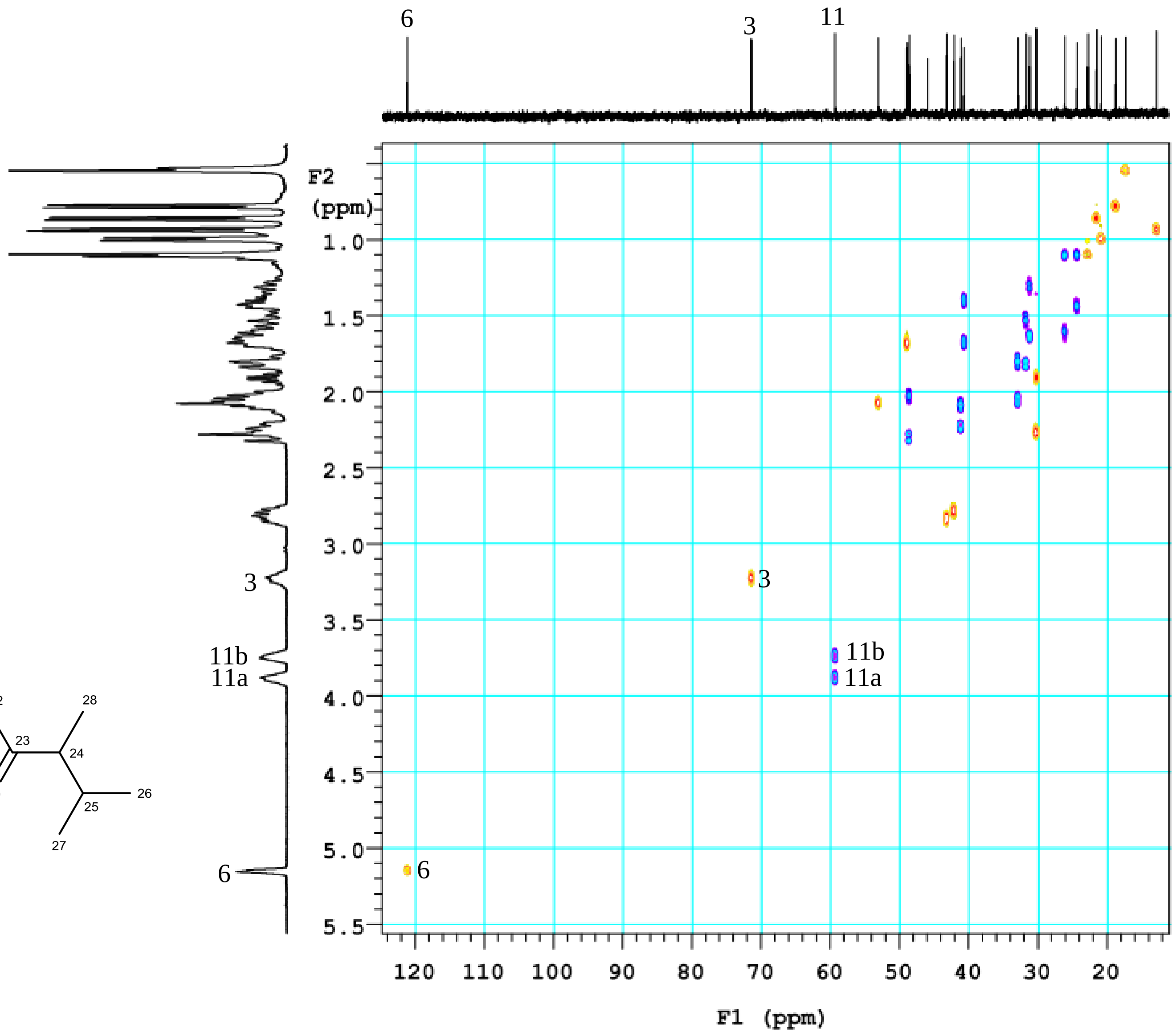

Figure S13. HSQC spectrum (400 MHz) of **5** in C_6D_6

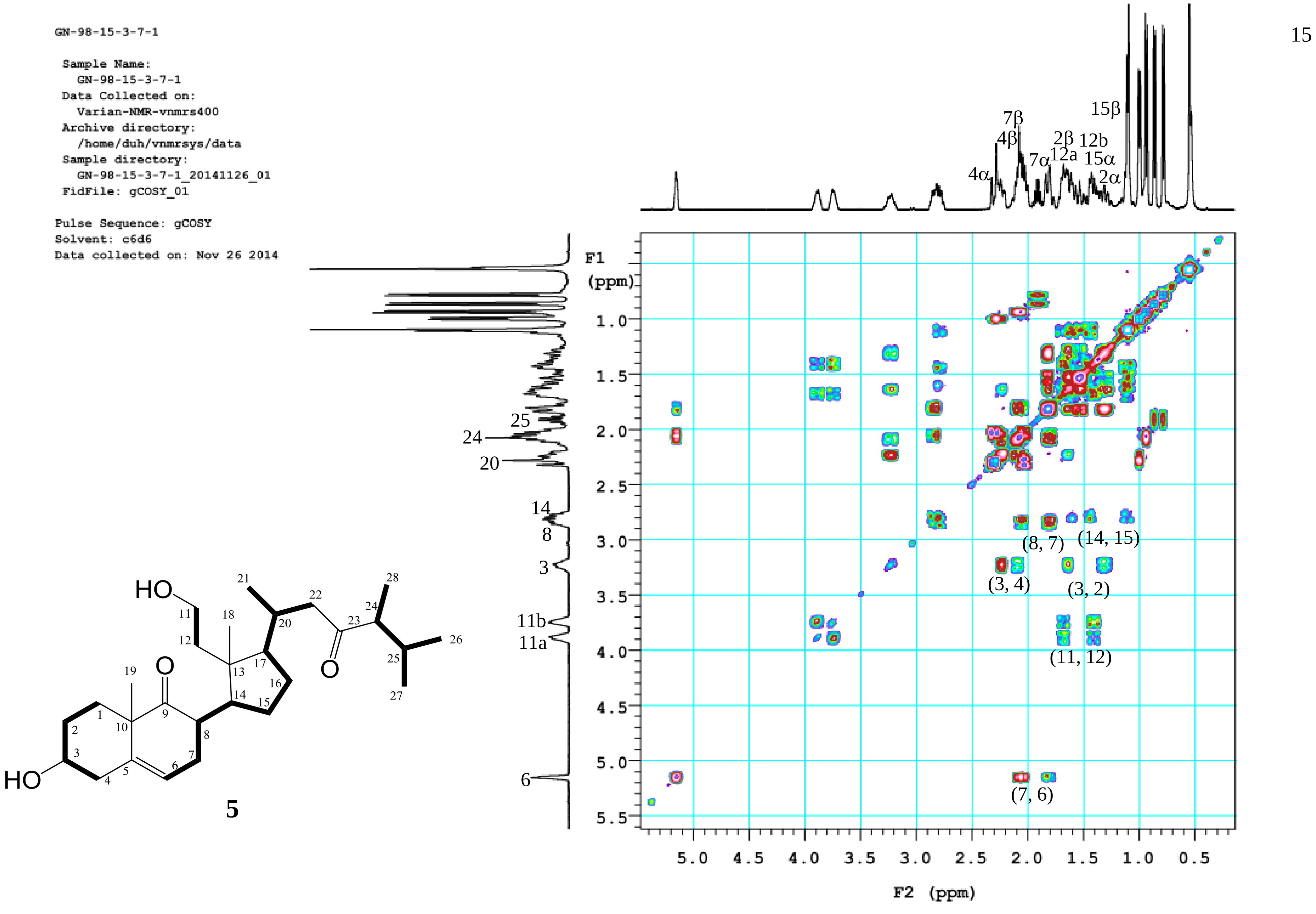

Figure S14. COSY spectrum (400 MHz) of **5** in C_6D_6

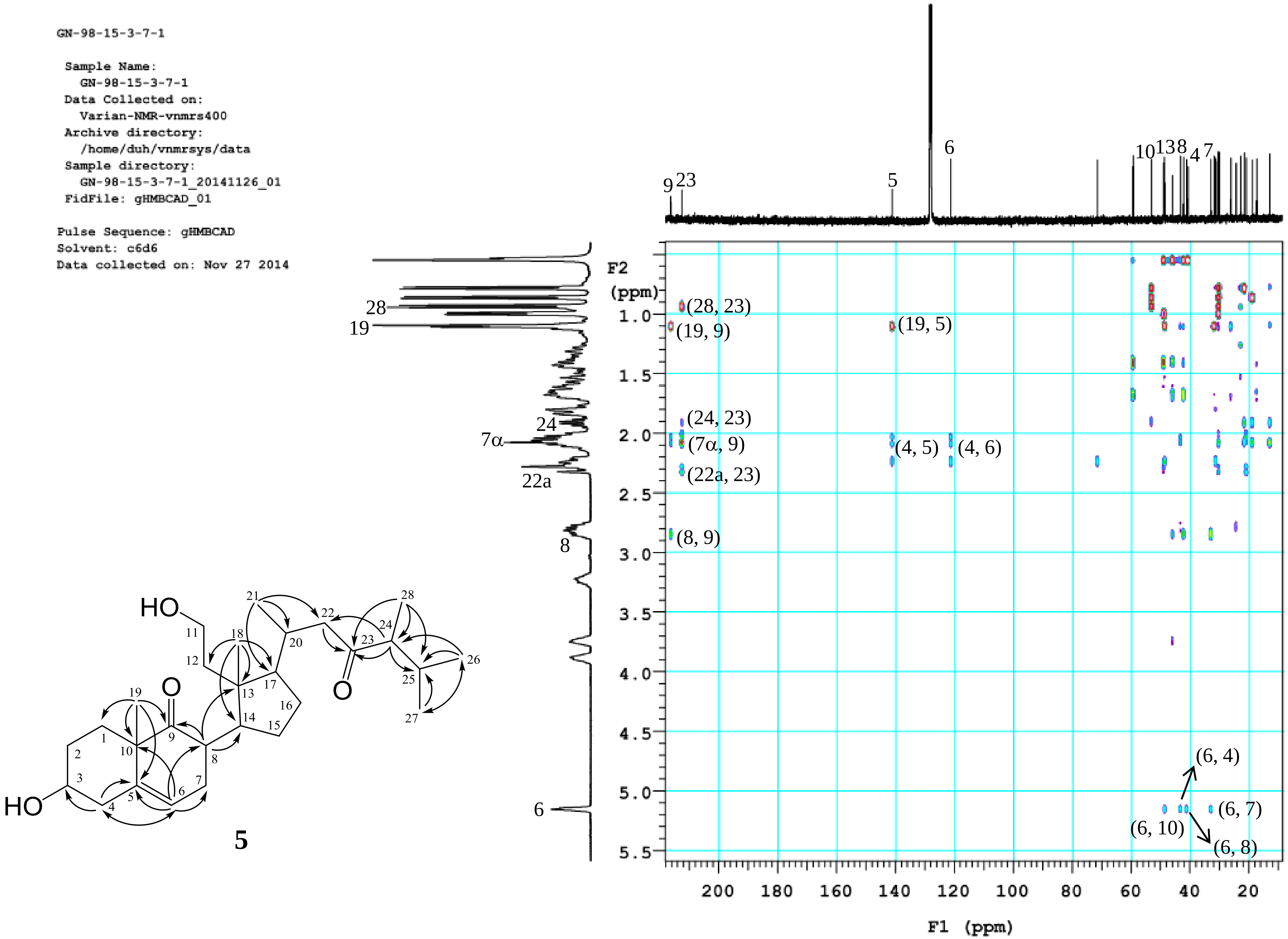

Figure S15. HMBC spectrum (400 MHz) of **5** in C_6D_6

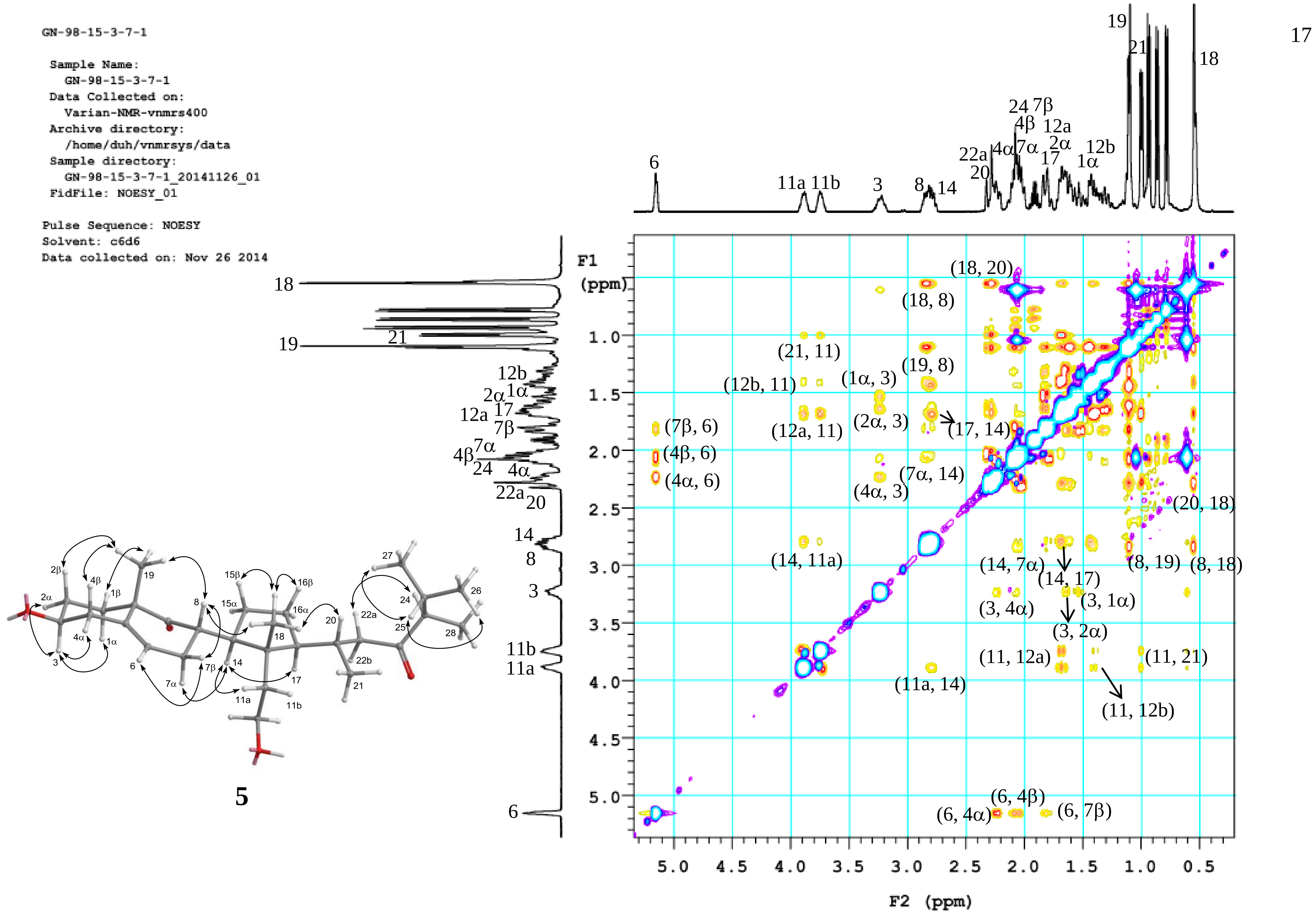

Figure S16. NOESY spectrum (400 MHz) of **5** in C_6D_6

Mass Spectrum SmartFormula Report

Analysis Info

Analysis Name	D:\Data\b4\gn9815374_000004.d	1/26/2015 2:35:27 PM	
Method	broadband first signal		
Sample Name	GN98-15-3-7-4	Instrument: FT-MS	solariX
Comment	ESI Positive		

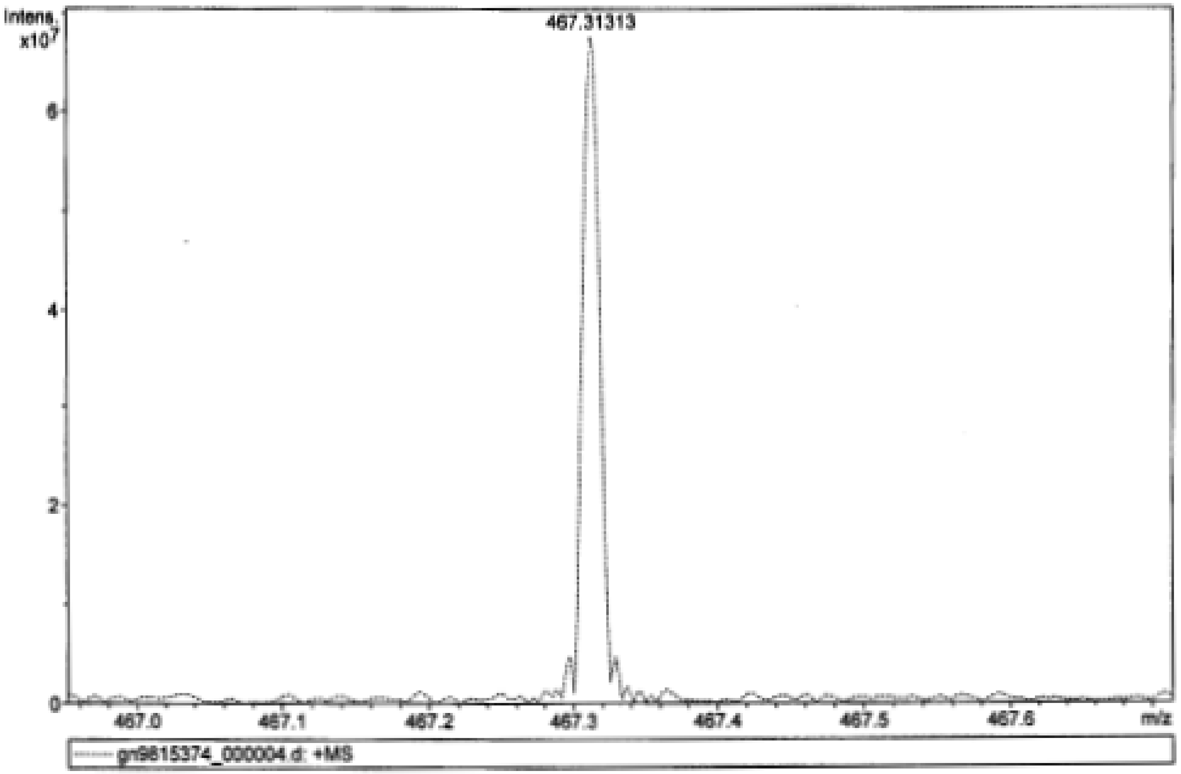

Meas. m/z	#	Formula	Score	m/z	err [mDa]	err [ppm]	mSigma	rdb	e⁻ Conf	N-Rule
467.31313	1	C 28 H 44 Na O 4	100.00	467.31318	0.06	0.12	10.0	6.5	even	ok

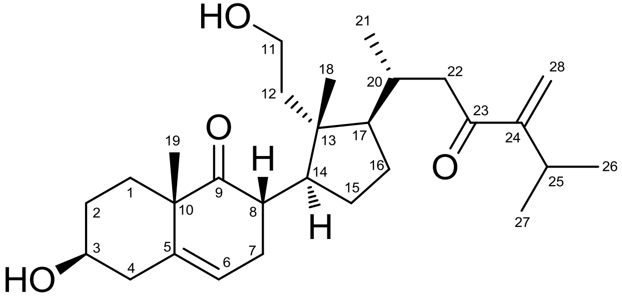

6

Figure S17. HRESIof **6**

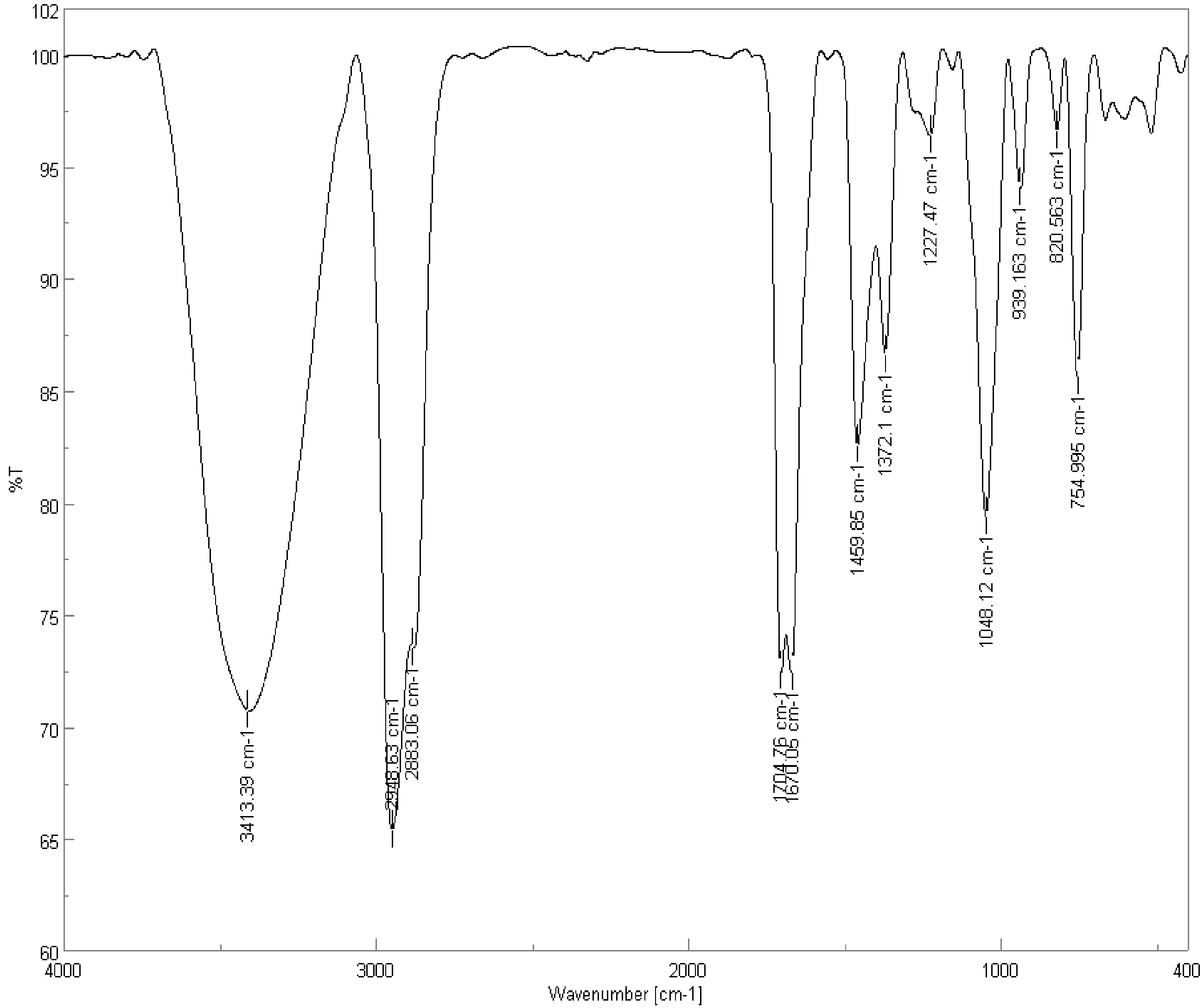

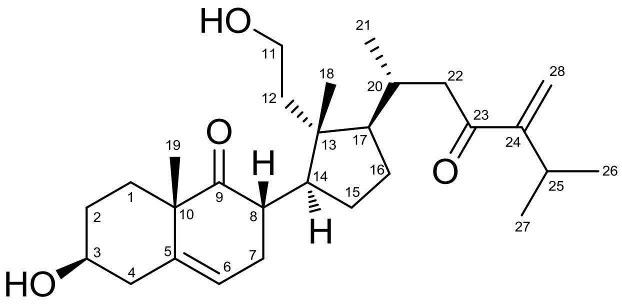

6

Figure S18. IR spectrum of **6**

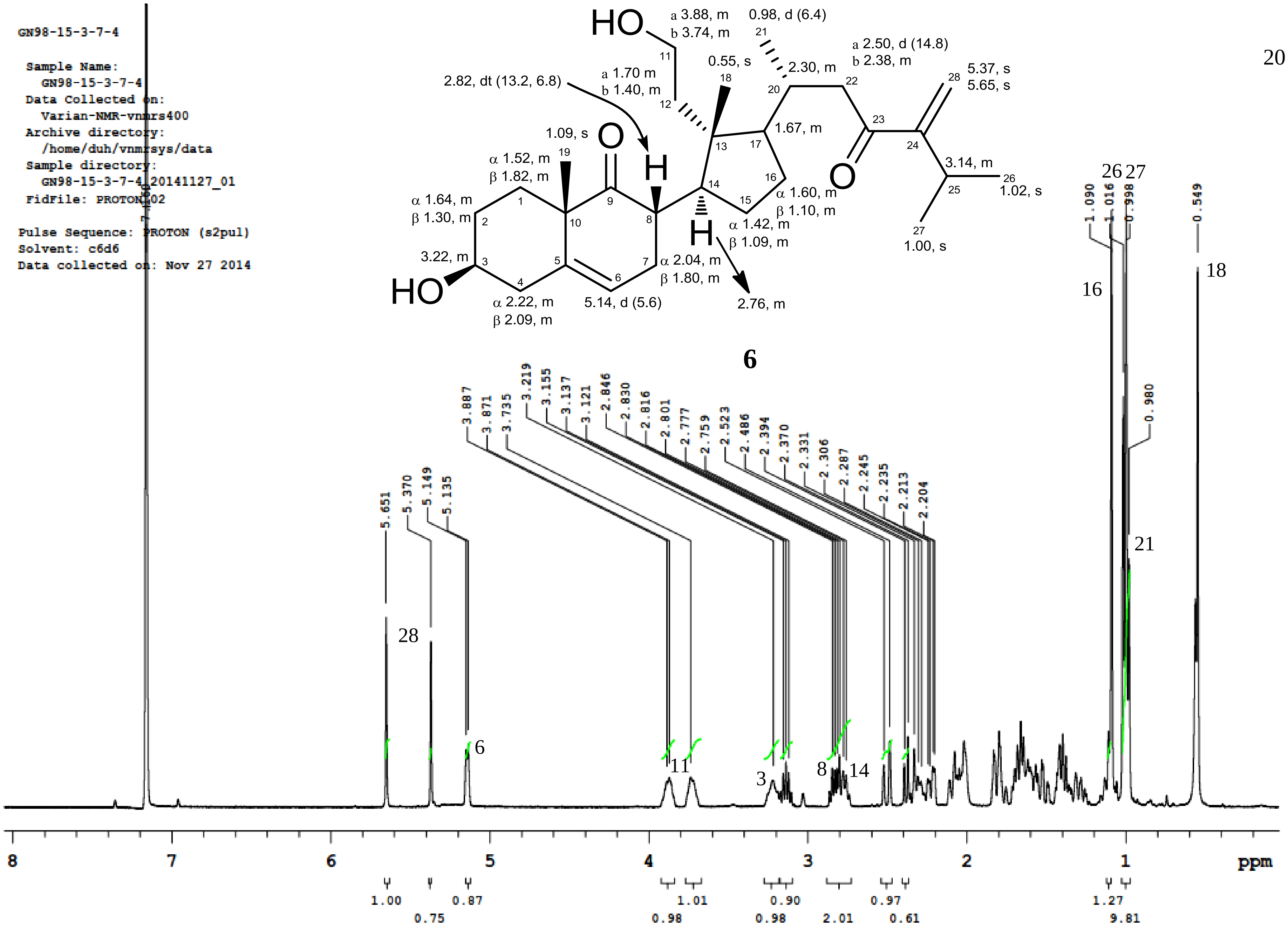

Figure S19. ^{1}H NMR spectrum (400 MHz) of **6** in C_6D_6

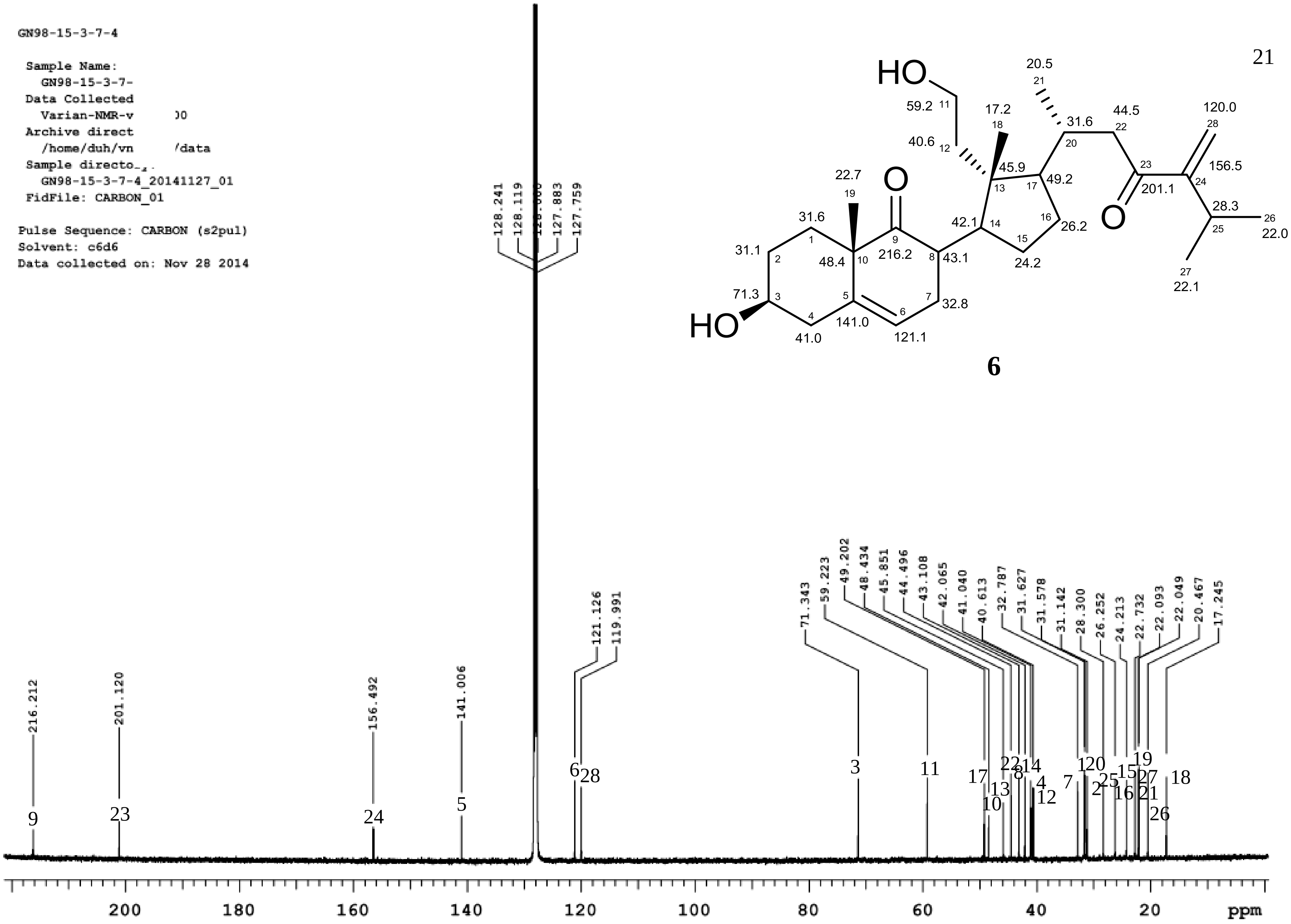

Figure S20. ^{13}CNMR spectrum (100 MHz) of **6** in C_6D_6

```
GN98-15-3-7-4

 Sample Name:
   GN98-15-3-7-4
 Data Collected on:
   Varian-NMR-vnmrs400
 Archive directory:
   /home/duh/vnmrsys/data
 Sample directory:
   GN98-15-3-7-4_20141127_01
 FidFile: gHSQCAD_01

Pulse Sequence: gHSQCAD
Solvent: c6d6
Data collected on: Nov 27 2014
```

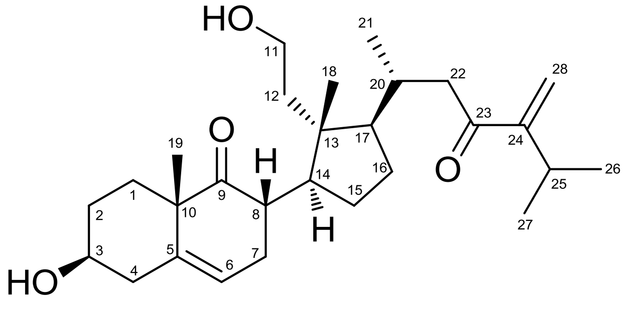

6

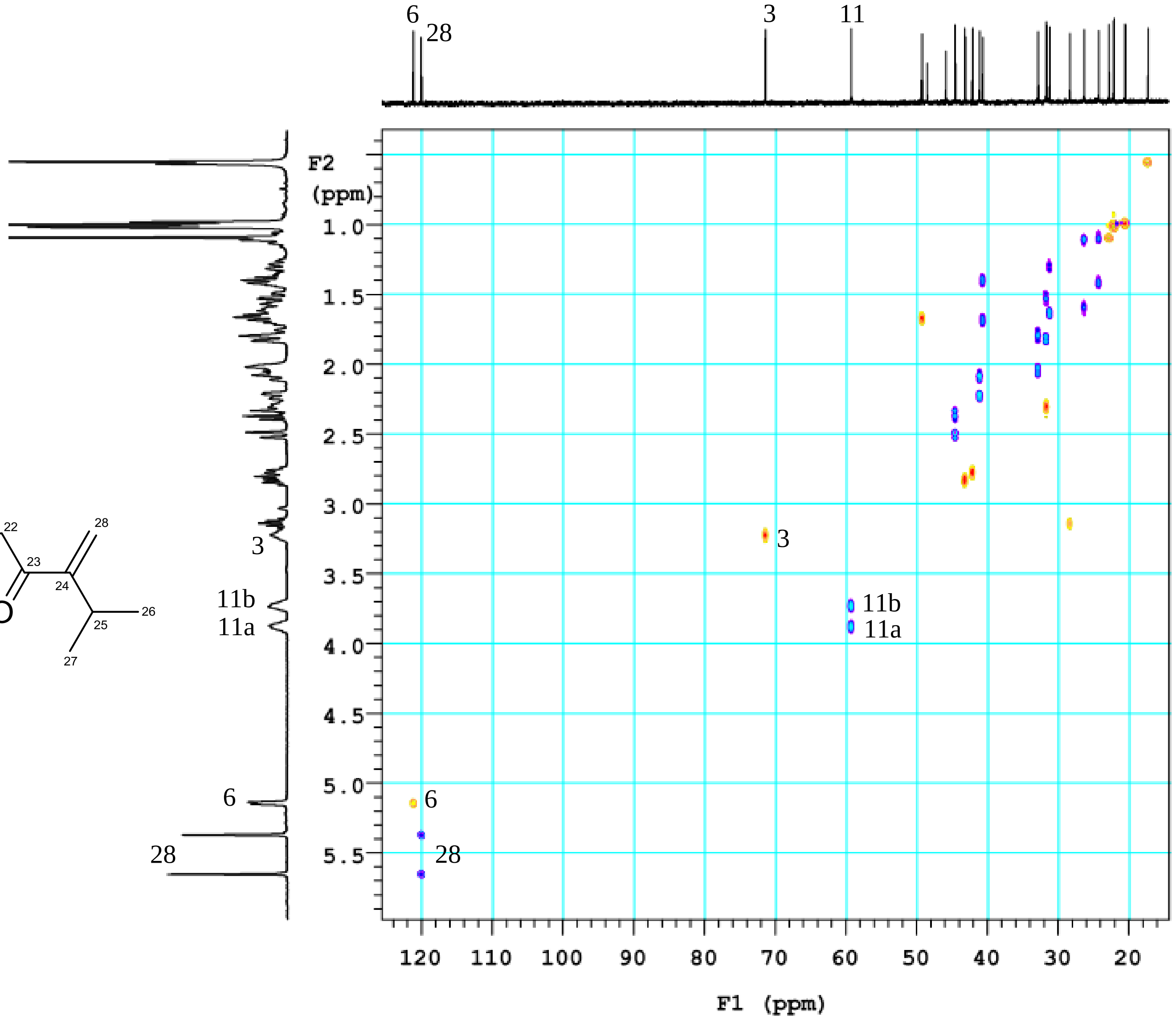

Figure S21. HSQC spectrum (400 MHz) of **6** in C_6D_6

```
GN98-15-3-7-4

 Sample Name:
   GN98-15-3-7-4
 Data Collected on:
   Varian-NMR-vnmrs400
 Archive directory:
   /home/duh/vnmrsys/data
 Sample directory:
   GN98-15-3-7-4_20141127_01
 FidFile: gCOSY_01

Pulse Sequence: gCOSY
Solvent: c6d6
Data collected on: Nov 27 2014
```

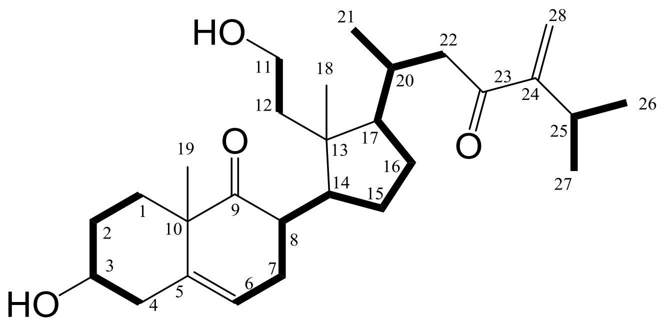

65

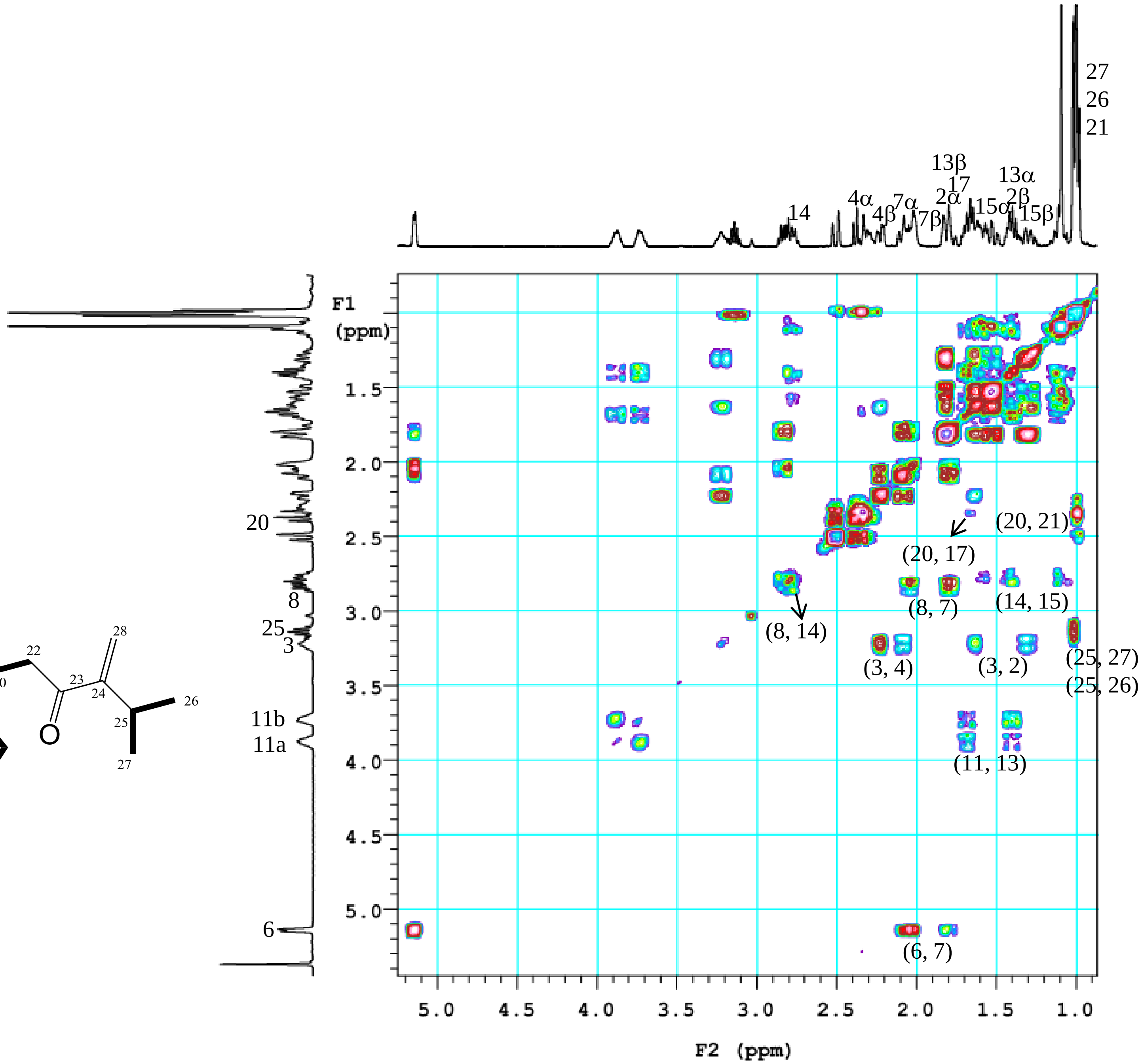

Figure S22. COSY spectrum (400 MHz) of **6** in C_6D_6

```
GN98-15-3-7-4

 Sample Name:
   GN98-15-3-7-4
 Data Collected on:
   Varian-NMR-vnmrs400
 Archive directory:
   /home/duh/vnmrsys/data
 Sample directory:
   GN98-15-3-7-4_20141127_01
 FidFile: gHMBCAD_01

Pulse Sequence: gHMBCAD
Solvent: c6d6
Data collected on: Nov 28 2014
```

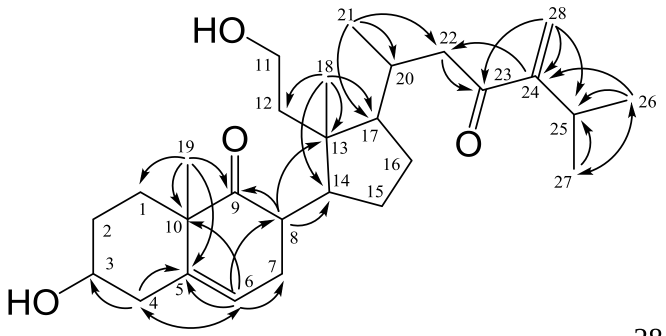

6

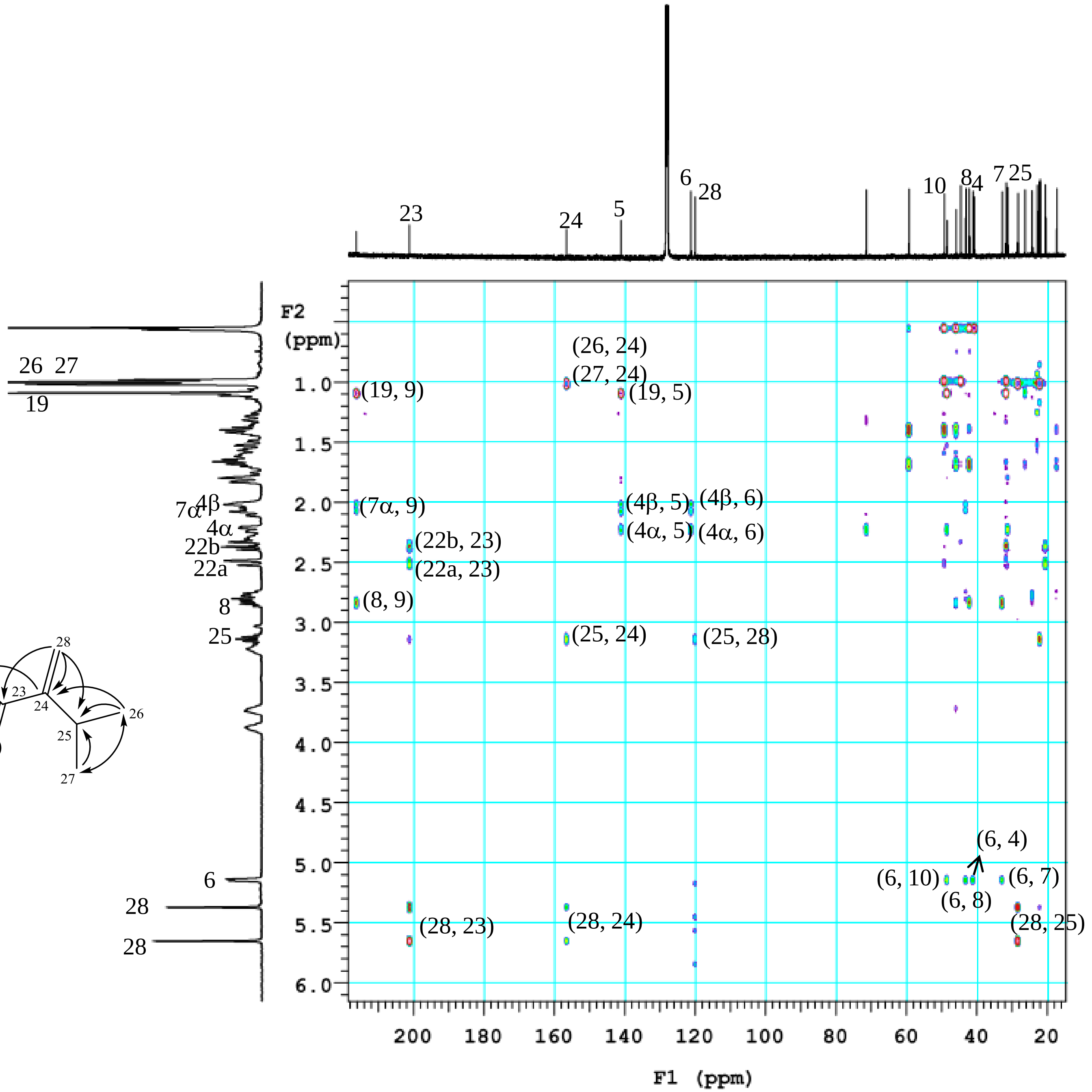

Figure S23. HMBC spectrum (400 MHz) of **6** in C_6D_6

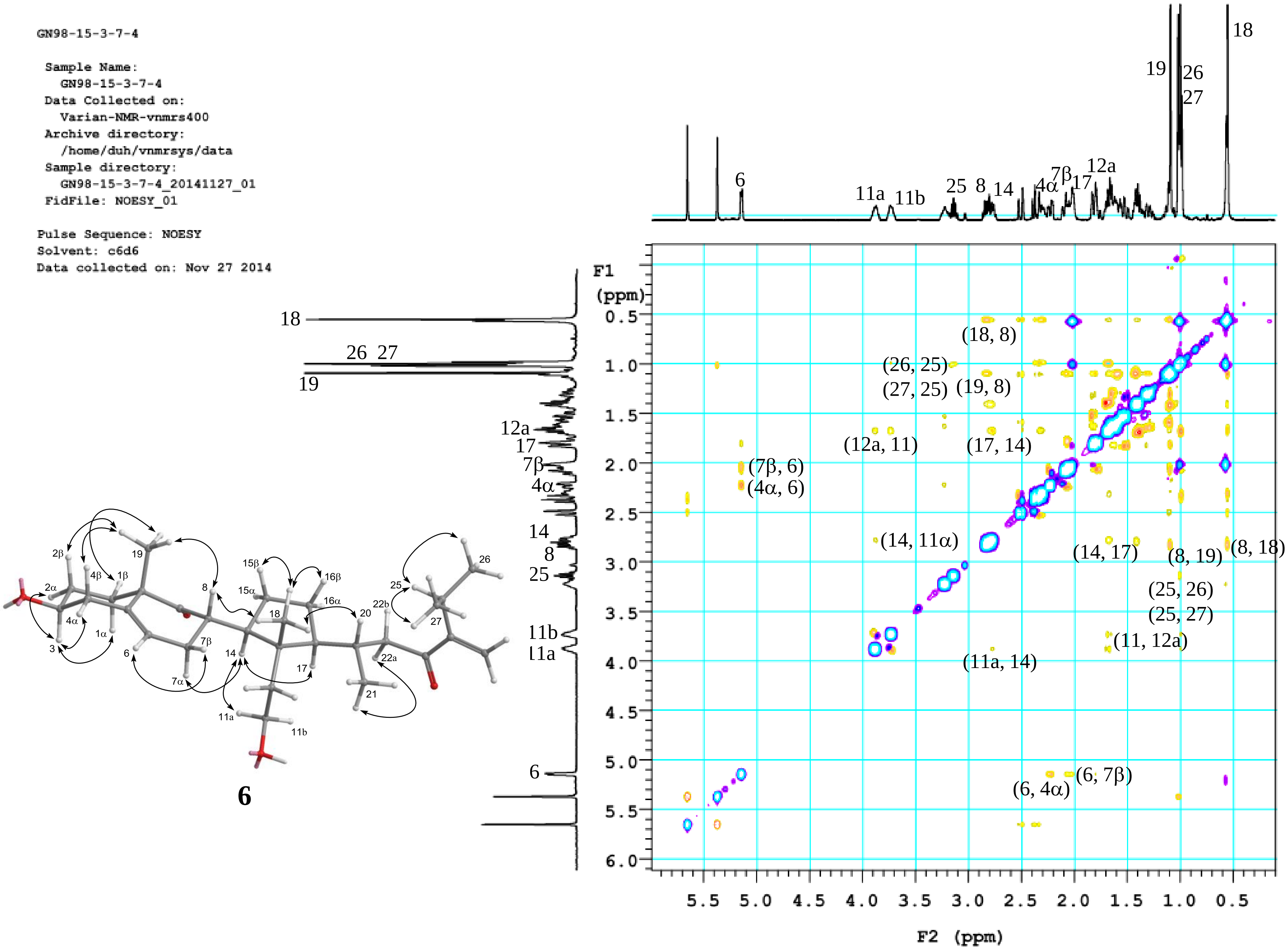

6

Figure S24. NOESY spectrum (400 MHz) of **6** in C_6D_6

Table S1. Evaluation of ED_{50} cytotoxicity of tested compounds against HEK293T cells

ID	Compound	M (g/mol)	MTT assay				High-content assay Nuclear count
			A549	HT29	P388	HEK293T-EGFP-UL76	
B	Bortezomib	384.243	25.7 nM	18.7 nM	1.6 nM	12.0 nM	24.3 nM
M	MG132	475.630	5.5 µM	6.8 µM	0.71 µM	1.2 µM	1.9 µM
1	Clavinflol C	372	>50 µg/ml (134.4 µM)	>50 µg/ml	>50 µg/ml	>25 µg/ml (67.2 µM)	6.1 µg/ml (16.4 µM)
2	Stolonidiol	336	3.9 µg/ml (11.6 µM)	>50 µg/ml (148.8 µM)	0.6 µg/ml (1.8 µM)	>25 µg/ml (74.4 µM)	>25 µg/ml
3	Stolonidiol-17-acetate	378	>50 µg/ml (132.3 µM)	>50 µg/ml	>50 µg/ml	>25µg/ml (66.1 µM)	19.6 µg/ml (51.9 µM)
4	Clavinflol B	372	>50 µg/ml (134.4 µM)	>50 µg/ml	>50 µg/ml	>25 µg/ml (67.2 µM)	21.3 µg/ml (57.3 µM)
5	3*β*,11-dihydroxy-24-methyl-9,11-secocholest-5-en-9,23-dione	446	>50 µg/ml (112.1 µM)	3.2 µg/ml (7.2 µM)	4.6 µg/ml (10.3 µM)	12.3 µg/ml (27.6 µM)	12.1 µg/ml (27.1 µM)
6	3*β*,11-dihydroxy-24-methylene-9,11-secocholest-5-en-9,23-dione	444	5.3 µg/ml (11.9 µM)	>50 µg/ml (112.6 µM)	4.8 µg/ml (10.8 µM)	>25 µg/ml (56.3 µM)	10.9 µg/ml (24.5 µM)
